# Clinical efficacy, safety evaluation, and mechanism of acetylcysteine in improving first-line anti-tuberculosis treatment of newly diagnosed pulmonary TB: an open-label, randomized, single-center trial

**DOI:** 10.3389/fmed.2026.1844872

**Published:** 2026-08-26

**Authors:** Huan Zhang, Ming Liu, Lu Shi, Xianlei Wang, Jiong Xie, Qingyan Ma

**Affiliations:** Three Tuberculosis Department, Hebei Chest Hospital, Shijiazhuang City, Hebei, China

**Keywords:** acetylcysteine, adverse reactions, clinical efficacy, conventional anti-TB drugs, newly treated pulmonary TB

## Abstract

**Objective:**

To investigate the clinical efficacy, safety and mechanism of N-acetylcysteine combined with conventional antituberculosis drugs in the treatment of newly diagnosed pulmonary tuberculosis.

**Methods:**

A total of 90 newly diagnosed pulmonary tuberculosis patients were randomly assigned to a Control group (*n* = 30), Aerosol NAC group (*n* = 30), or Oral NAC group (*n* = 30). The Control group received standard HRZE therapy, whereas the Aerosol NAC and Oral NAC groups received additional nebulized N-acetylcysteine (300 mg twice daily) or oral N-acetylcysteine (600 mg once daily), respectively. Clinical efficacy, sputum conversion, St. George’s Respiratory Questionnaire (SGRQ) scores, liver function indices, oxidative stress biomarkers, inflammatory markers, and adverse events were assessed over 3 months. Data were analyzed using one-way ANOVA, repeated-measures ANOVA, Chi-square tests, and Bonferroni-adjusted *post hoc* analyses.

**Results:**

Treatment successful response rates were significantly higher in the Aerosol NAC group (96.67%) and Oral NAC group (93.33%) than in the Control group (70.00%) (*p* < 0.05). At 3 months, sputum conversion rates reached 93.33 and 96.67% in the Aerosol NAC and Oral NAC groups, respectively, compared with 73.33% in the Control group (*p* < 0.05). Both NAC groups demonstrated significantly lower SGRQ scores, improved liver function parameters (AST, ALT, TBIL, and GGT), higher antioxidant enzyme levels (SOD and GSH-Px), lower MDA concentrations, and reduced inflammatory markers (IFN-*γ*, PCT, and IL-4) compared with the Control group (all *p* < 0.05). The incidence of adverse reactions was lower in both NAC groups (10.0%) than in the Control group (40.0%) (p < 0.05).

**Conclusion:**

Adjunctive N-acetylcysteine, administered either by aerosol inhalation or orally, significantly improved treatment outcomes, accelerated sputum conversion, reduced oxidative stress and inflammation, protected liver function, and decreased adverse events in patients with newly diagnosed pulmonary tuberculosis.

## 1 Introduction

Tuberculosis (TB) is an infectious disease caused by *Mycobacterium tuberculosis* (MTB) infection, which is mainly transmitted through the respiratory tract, and its mortality rate ranks first among the causes of single infectious diseases since 2007 Although early detection and early treatment greatly improve the cure rate of TB, TB is still one of the main reasons threatening public health in many countries due to the acceleration of population mobility and the emergence of drug-resistant strains of MTB. According to the 2020 Global (1). TB Report released by WHO, the number of new TB cases in the world in 2019 was estimated to be 10 million, of which there were about 833,000 new TB patients in our country (2). As China is a country with a high burden of TB, there are a large number of carriers and cases. The epidemic situation of TB is not optimistic, so we should actively carry out the prevention and control of TB. Early diagnosis and early treatment are a successful way to curb the spread of TB and reduce the fatality rate of TB.

At present, chemotherapy is mainly used to treat TB, among which the commonly used anti-TB drugs include rifampicin (RFP), isoniazid (INH), ethambutol (EMB), pyrazinamide (PZA), streptomycin (SM), levofloxacin (LOFX), amikacin (AMK), capreomycin (CPM), p-amino salicylic acid (PAS) and so on (3). The principles of early treatment, whole-course treatment, regular treatment, moderate treatment, and combined treatment should be followed by patients with pulmonary TB. The standard short-course chemotherapy regimen promoted by WHO for drug-sensitive TB is to take rifampicin (RFP), isoniazid (INH), ethambutol (EMB) and pyrazinamide (PZA) in the first 2 months, and then continue to take RFP and INH for at least 4 months (consolidation) (4). Among these drugs, INH, PZA and EMB are specific drugs against mycobacteria, while RFP is a broad-spectrum antibiotic. INH is activated by the bacterial internal hydrogen peroxide-peroxidase KatG and acts on the type II fatty acid synthase system of mycobacteria, thereby inhibiting the production of sensitive bacterial mycolic acid by the organism (1). PZA can penetrate MTB-containing phagocytes and convert them into pyrazinoic acid inside MTB, thereby exerting an antibacterial effect (5). In the meanwhile, the chemical structure of PZA is similar to that of nicotinamide, which can interfere with the oxygen utilization of MTB by interfering with the dehydrogenation process of dehydrogenase, thus interfering with the normal metabolic process of MTB and ultimately leading to the death of MTB (6). EMB can inhibit the reproduction of MTB by disrupting the RNA synthesis process of MTB cells (7). There are gram-positive and gram-negative bacteria that are susceptible to the broad-spectrum antibiotic RFP that belongs to the rifamycin family (8). RFP can mainly act on the *β* subunit of MTB RNA polymerase and inhibits the growth of MTB by affecting the transcription of MTB, to kill MTB and fight TB.

In-depth studies have found that long-term use of quadruple anti-TB treatment is more toxic to the liver and kidneys, so it is vital to find a safe and successful treatment method. Acetylcysteine is a derivative of cysteine N-acetylation and is a mucus solubilizer. It can quickly and strongly dissolve mucus and thick mucus secretions, reduce the viscosity of sputum, promote the elimination of TB bacteria in the respiratory tract and inhibit the fixed value of pathogen. Acetylcysteine itself can also provide nucleophilic free sulfhydryl groups and play a direct antioxidant role (9, 10), promote respiratory barrier repair and anti-inflammation, and improve or maintain lung function in patients. In addition, NAC can also convert oxidized glutathione into reduced glutathione, enhance its antioxidant capacity, and achieve the purpose of preventing cell damage (11, 12). The main clinical modes of administration of NAC are nebulized inhalation and oral administration. Acetylcysteine nebulized inhalation works by nebulizing the gas and inhaling it directly into the respiratory tract. It enters the terminal bronchi and alveoli with deep inhalation, acts on localized lesions, improves pulmonary circulation, and promotes the formation of alveolar surfactant and the absorption of gas and drug (13). Previous studies have shown that the combination of NAC and anti-TB drugs when treating pulmonary TB can improve the oxidation/antioxidation imbalance (14). It is reported that NAC adjuvant treatment of pulmonary TB can enhance the clinical symptoms and life quality of patients with chronic fibrous cavity pulmonary TB (15). However, newly diagnosed pulmonary TB is treated with NAC associated with conventional anti-TB drugs, but little is known about their effectiveness and safety. There is no consensus on which mode of administration is the best. Against this background, it is necessary to carry out further research to clarify the mechanism of NAC and optimize the choice of administration mode for this kind of patient. Based on the above problems, this study used anti-TB drugs associated with the NAC regimen to treat patients with newly diagnosed pulmonary TB. From a clinical point of view, we analyzed whether NAC as a routine adjuvant treatment of pulmonary TB could more quickly promote focus healing, and reduce the recurrence rate and liver injury and other adverse reactions at first. Further exploration is through anti-oxidation and anti-inflammatory mechanisms to achieve the expected clinical effect of pulmonary TB treatment.

## 2 Patients and methods

### 2.1 Study design and setting

This was a randomized, open-label, parallel-group controlled trial conducted at the Three Tuberculosis Department, Hebei Chest Hospital, Shijiazhuang City, Hebei Province, China, from April 2021 to April 2022. A total of 90 patients with newly diagnosed pulmonary tuberculosis were enrolled and randomly assigned in a 1:1:1 ratio to the Control group (standard HRZE therapy plus sham nebulization, *n* = 30), Aerosol NAC group (standard HRZE therapy plus nebulized N-acetylcysteine, *n* = 30), or Oral NAC group (standard HRZE therapy plus oral N-acetylcysteine, *n* = 30). The trial evaluated the clinical efficacy, safety, and mechanistic effects (oxidative stress and inflammation) of adjunctive N-acetylcysteine administered by two routes.

The sample size of 30 patients per group was determined on the basis of feasibility for a single-center study conducted during the COVID-19 pandemic and by reference to previous adjunctive NAC trials in pulmonary TB and COPD (typical arm sizes 20–40 patients). No formal *a priori* power calculation was performed for a pre-specified primary endpoint. Post-hoc power analysis indicated that the study had >80% power (*α* = 0.05, two-sided) to detect the observed difference in treatment success rate (70% vs. 95%).

### 2.2 Participant recruitment and sampling

Consecutive patients presenting with clinical features suggestive of pulmonary tuberculosis (afternoon low-grade fever, cough, expectoration with thick sputum) were screened in the outpatient and inpatient services. Ninety patients who met the eligibility criteria and provided written informed consent were enrolled. Baseline demographic and clinical characteristics (age, sex, body weight, BMI, disease duration, and education level) were comparable among the three groups (*p* > 0.05); detailed data are presented in the Results section.

### 2.3 Randomization and allocation concealment

**Figure 1:**
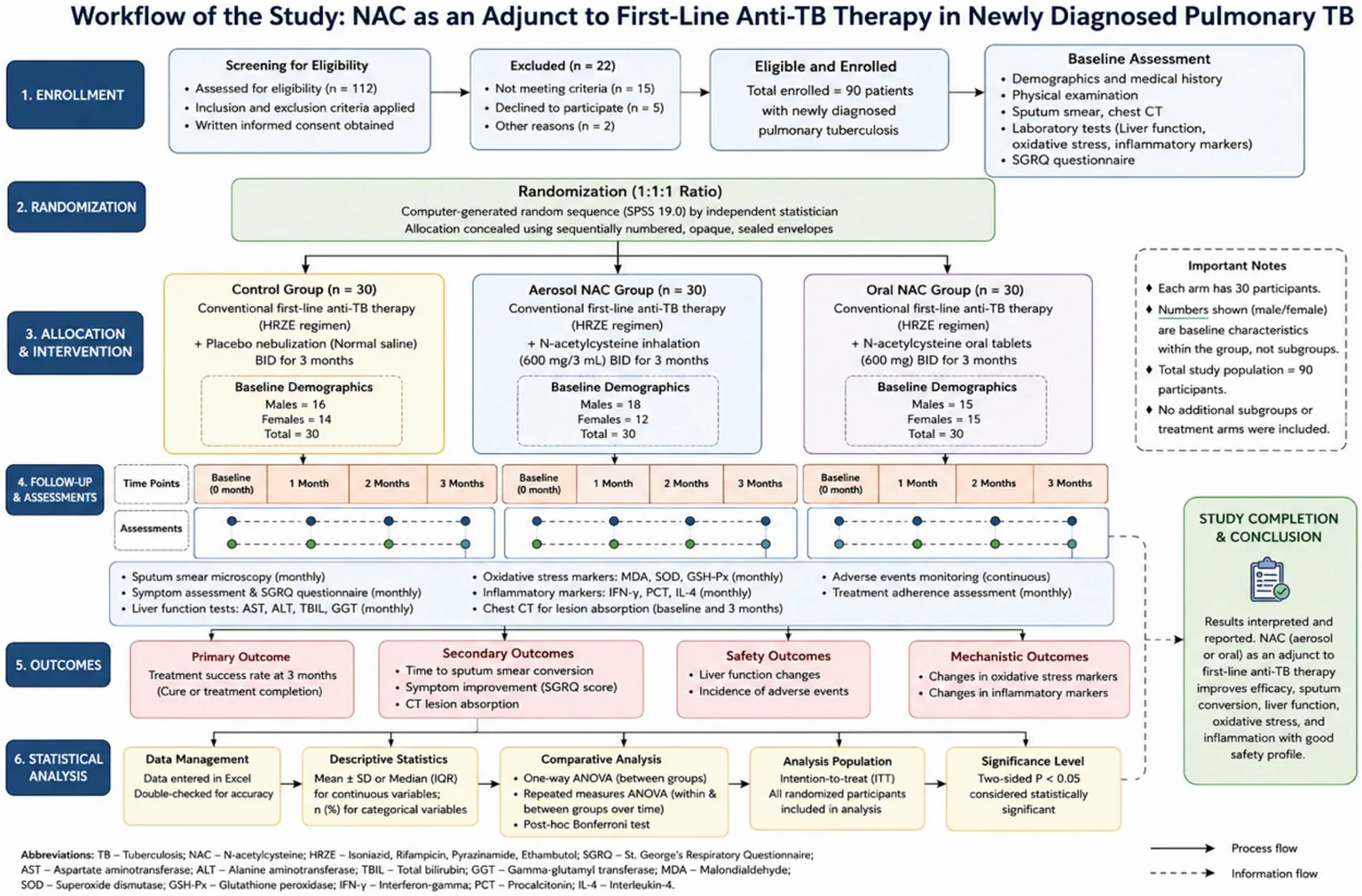
Study workflow and design.

Patients were randomly allocated to one of the three groups in a 1:1:1 ratio using a computer-generated random number sequence created in SPSS 19.0 by an independent statistician who was not involved in patient recruitment, treatment administration, or outcome assessment. Allocation concealment was achieved using sequentially numbered, opaque, sealed envelopes prepared in advance. The envelopes were opened only after written informed consent had been obtained and baseline evaluations completed. Group assignment was recorded on a separate log sheet that was inaccessible to the clinical team until the end of the study. The study workflow is illustrated in Figure 1.

### 2.4 Blinding

Because the interventions involved different routes of administration (aerosol inhalation versus oral administration), blinding of participants and treating clinicians was not feasible. Therefore, the study was conducted as an open-label randomized controlled trial. However, laboratory personnel, radiologists interpreting chest CT examinations, and investigators responsible for statistical analyses remained blinded to treatment allocation to reduce the risk of assessment bias.

Flow diagram illustrating the study design, participant enrollment, randomization, interventions, follow-up assessments, outcome measures, and statistical analysis. A total of 90 newly diagnosed pulmonary tuberculosis patients were screened, enrolled, and randomly allocated in a 1:1:1 ratio to three groups: Control (standard HRZE therapy plus saline nebulization, *n* = 30), Aerosol NAC (standard HRZE therapy plus nebulized N-acetylcysteine, *n* = 30), and Oral NAC (standard HRZE therapy plus oral N-acetylcysteine, *n* = 30). Participants were followed for 3 months with serial evaluations of clinical efficacy, sputum smear conversion, health-related quality of life (SGRQ), liver function parameters, oxidative stress biomarkers (MDA, SOD, and GSH-Px), inflammatory markers (IFN-*γ*, PCT, and IL-4), and treatment-related adverse events. Statistical analyses included one-way ANOVA, repeated-measures ANOVA, and Bonferroni-adjusted *post hoc* comparisons.

### 2.5 Eligibility criteria

#### 2.5.1 Inclusion criteria

(1) All confirmed by X-ray, CT, and sputum acid-fast staining. The clinical manifestations are low-grade fever in the afternoon, cough, expectoration, and thick sputum, which conform to the diagnostic criteria of pulmonary TB in the “Chinese Expert Consensus on Pathological Diagnosis of TB” (16), and all were newly treated pulmonary TB patients; (2) No application in the past 0.5 years Antioxidants such as immunosuppressants and vitamin C. (3) The informed consent form was signed by all patients before the test was conducted.

#### 2.5.2 Exclusion criteria

(1) Those with a history of allergy to acetylcysteine or various drugs or with an allergic constitution; (2) Those with bronchial asthma; (3) Those with severe liver, kidney, heart, and hematopoietic system diseases; (4) Those with active gastric ulcers (5) Pregnant women and lactating women; (6) Those with poor treatment compliance; (7) Those with severe mental illness and cognitive impairment; (8) Those who dropped out of this study halfway through.

### 2.6 Interventions

Participants received standard first-line anti-tuberculosis chemotherapy with or without adjunctive N-acetylcysteine (NAC) according to group allocation. Detailed treatment regimens, routes of administration, dosages, frequencies, and treatment durations are summarized in Table 1.

**Table 1:** Treatment regimens and dosages administered to study participants.

Group	Intervention	Route of administration	Dosage	Frequency	Duration
Control Group (*n* = 30)	Standard first-line anti-TB chemotherapy	Oral	Isoniazid 300 mg, Rifampicin 450–600 mg, Pyrazinamide 1,500 mg, Ethambutol 750 mg*	Once daily	3 months
Aerosol NAC Group (*n* = 30)	Standard anti-TB chemotherapy + NAC	Aerosol inhalation	NAC 600 mg	Twice daily	3 months
Oral NAC Group (*n* = 30)	Standard anti-TB chemotherapy + NAC	Oral	NAC 600 mg	Twice daily	3 months

*Dosages of anti-tuberculosis drugs were adjusted according to body weight and national treatment guidelines where applicable.

NAC, N-acetylcysteine.

### 2.7 Outcome measures

#### 2.7.1 Clinical treatment efficacy

Clinical efficacy was evaluated after 3 months of treatment according to the Belknap (31). *A remarkably successful outcome was defined as >50% lesion absorption* on chest CT, marked improvement or complete disappearance of clinical symptoms such as cough and fever, and negative sputum smear results. A successful outcome was defined as lesion absorption of ≥20% but <50%, improvement in clinical symptoms, and either negative or positive sputum smear results. An unsuccessful outcome was defined as lesion absorption <20%, persistence or worsening of symptoms, and positive sputum smear results. The treatment success rate was calculated as follows:

Treatment Success Rate (%) = [(Number of Remarkably Successful Cases + Number of Successful Cases) ÷ Total Number of Cases] × 100.

#### 2.7.2 Health status and quality-of-life assessment

Patient prognosis, health status, and quality of life were assessed at 1, 2, and 3 months after treatment using the St. George’s Respiratory Questionnaire (SGRQ). The questionnaire consists of 50 items divided into three domains: symptoms, activity, and impact. The symptoms domain evaluates the frequency and severity of respiratory symptoms, the activity domain assesses physical activity limitations due to breathlessness, and the impact domain measures the social and psychological consequences of respiratory disease. Scores were calculated using the weighted scoring method recommended in the SGRQ manual. Each domain score was expressed as a percentage of the maximum possible score, resulting in a range of 0–100, with lower scores indicating better health-related quality of life.

#### 2.7.3 Sputum smear conversion

Sputum smear conversion was evaluated at 1, 2, and 3 months after treatment. Three sputum specimens were collected from each patient at each assessment point and examined using Peng’s cup acid-fast staining method according to the Rules for Bacteriological Examination in Tuberculosis Diagnosis. The sputum conversion rate was calculated as the proportion of patients with negative sputum smear results among the total number of patients in each group.

### 2.8 Laboratory assessments

Blood biochemical analyses were performed using a Beckman Coulter AU680 automatic biochemical analyzer. Liver function was assessed by measuring aspartate aminotransferase (AST), alanine aminotransferase (ALT), total bilirubin (TBIL), and gamma-glutamyl transferase (GGT). Oxidative stress biomarkers included superoxide dismutase (SOD), glutathione peroxidase (GSH-Px), and malondialdehyde (MDA). SOD and GSH-Px activities were measured using the xanthine oxidase method, whereas MDA levels were determined using the thiobarbituric acid method.

Serum inflammatory markers, including interferon-gamma (IFN-*γ*), procalcitonin (PCT), and interleukin-4 (IL-4), were measured using a Roche Cobas E411 electrochemiluminescence immunoassay analyzer and double-antibody sandwich ELISA kits supplied by Shanghai Jingkang Bioengineering Co., Ltd. All laboratory measurements were performed at 1, 2, and 3 months after treatment according to the manufacturers’ instructions.

### 2.9 Safety evaluation

Treatment-related adverse events were monitored throughout the study period. Recorded adverse reactions included nausea and vomiting, dizziness, diarrhea, skin rash, and fever. The incidence of adverse events was calculated and compared among the three study groups.

### 2.10 Statistical analysis

The SPSS19.0 software (IBM Corp., Armonk, NY, USA) was adopted for data analysis, in which the measurement data with normal distribution and uniform variance were expressed as (±s). One-way ANOVA was used for comparison among the three groups. And repeated measurement data were repeated measurement ANOVA. We compared the two groups by using the Chi-square test and expressed the counting data as [*n* (%)]. In terms of statistical significance, the difference was remarkable (*p <* 0.05). One-way ANOVA with post-hoc Bonferroni correction was used for between-group comparisons at each time point. For repeated-measures data (SGRQ scores, liver function indices, SOD, MDA, GSH-Px, IFN-*γ*, PCT, and IL-4), repeated-measures ANOVA was performed with time, group, and time × group interaction as factors. When the time × group interaction was statistically significant, simple-effects analysis with Bonferroni adjustment was conducted at each time point. A two-sided *p* < 0.05 was considered significant.

### 2.11 Ethics approval and informed consent

This study was approved by the Ethics Committee of Hebei Chest Hospital, Shijiazhuang, Hebei Province, China (Approval No.: HCH-2021-042). The study was conducted in accordance with the Declaration of Helsinki and relevant national guidelines for human research. Written informed consent was obtained from all participants before enrollment. All participants received detailed information regarding the study objectives, procedures, potential risks, and expected benefits and were informed of their right to withdraw from the study at any time without prejudice to their medical treatment.

## 3 Results

### 3.1 Baseline characteristics of participants

Table 1 summarizes the baseline demographic and clinical characteristics of the study participants. A total of 90 patients were enrolled and randomized equally into the Control group (*n* = 30), Aerosol NAC group (*n* = 30), and Oral NAC group (*n* = 30). There were no statistically significant differences among the three groups with respect to age, sex distribution, body weight, body mass index, disease duration, educational level, or baseline clinical characteristics (all *p* > 0.05), indicating that the groups were comparable before treatment initiation (Table 2).

**Table 2:** Baseline demographic and clinical characteristics of study participants.

Characteristic	Control group (*n* = 30)	Aerosol NAC group (*n* = 30)	Oral NAC group (*n* = 30)	*p*-value
Age (years), mean ± SD	45.8 ± 11.2	46.3 ± 10.7	44.9 ± 12.1	0.874
Male, *n* (%)	16 (53.3)	18 (60.0)	15 (50.0)	0.731
Female, *n* (%)	14 (46.7)	12 (40.0)	15 (50.0)	0.731
Body weight (kg), mean ± SD	61.4 ± 8.5	62.1 ± 7.9	60.8 ± 8.3	0.812
Body mass index (kg/m^2^), mean ± SD	22.4 ± 2.6	22.7 ± 2.4	22.2 ± 2.8	0.768
Disease duration (months), mean ± SD	3.8 ± 1.2	4.0 ± 1.1	3.9 ± 1.3	0.845
Primary school or below, *n* (%)	8 (26.7)	7 (23.3)	9 (30.0)	0.881
Secondary school, *n* (%)	14 (46.7)	15 (50.0)	13 (43.3)	0.892
College or above, *n* (%)	8 (26.7)	8 (26.7)	8 (26.7)	1.000
Baseline sputum smear positive, *n* (%)	30 (100)	30 (100)	30 (100)	NA

Data are presented as mean ± standard deviation (SD) or number (%). p-values were calculated using one-way analysis of variance (ANOVA) for continuous variables and the Chi-square test for categorical variables. No statistically significant differences were observed among the three groups at baseline (all *p* > 0.05).

### 3.2 Comparison of therapeutic effects among the three groups

There was no loss to follow-up. All 90 randomized patients (100%) completed the full 3-month treatment course and all scheduled assessments at 1, 2, and 3 months. No patient met exclusion criterion.

**Figure 2:**
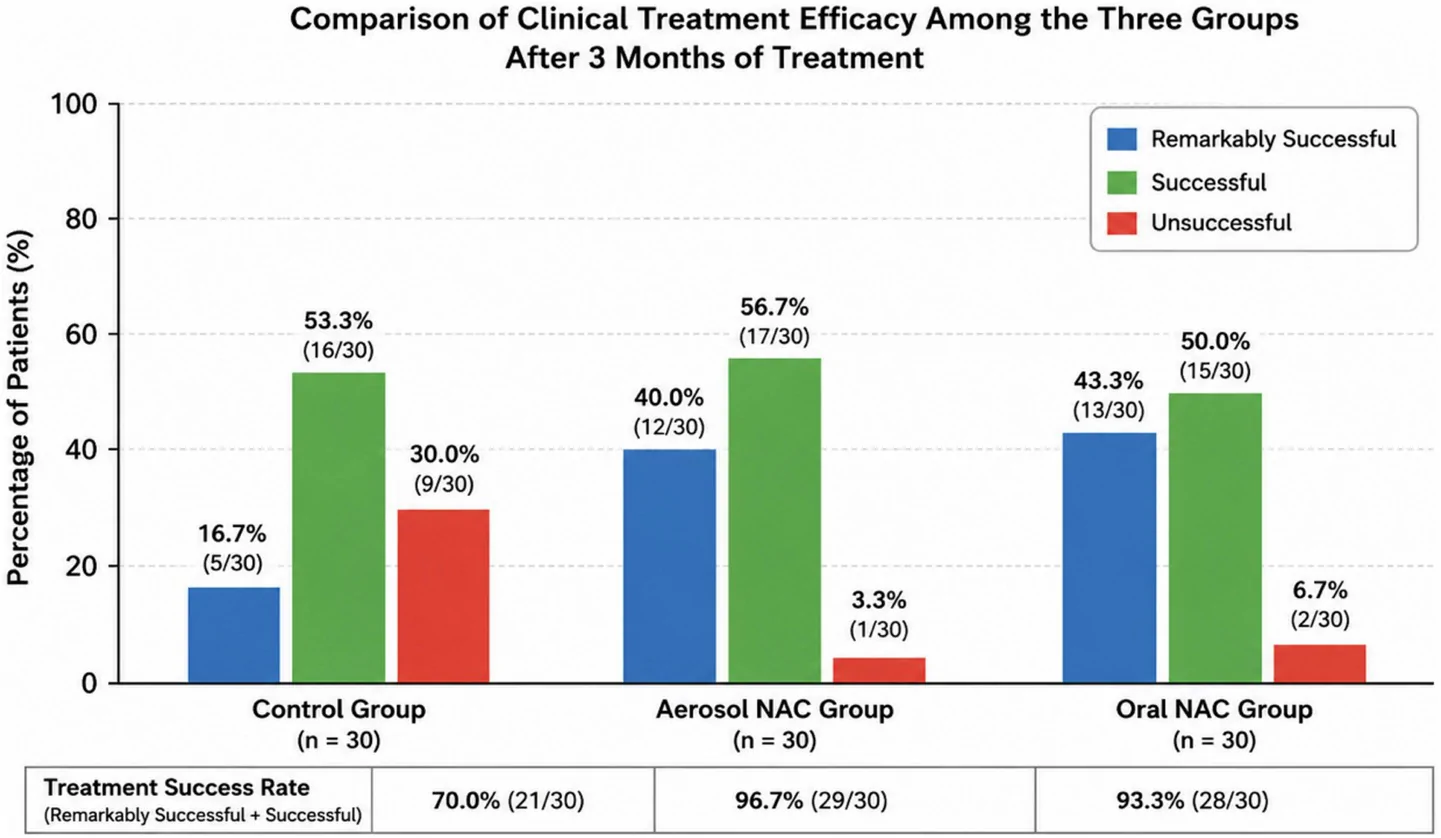
Comparison of clinical treatment efficacy among the three study groups after 3 months of treatment.

In the control group, 5 people were extremely successful, 16 people were successful, and 9 people were unsuccessful, and the success rate was 70%. In the Aerosol NAC group, 12 people were extremely successful, 17 people were successful, and 1 person was unsuccessful, and the treatment success rate was 96.67%. In the oral NAC group, 13 people were extremely successful, 15 people were successful, and 2 people were unsuccessful, and the treatment success rate was 93.33%. The success rates of the Aerosol NAC group and the oral NAC group were remarkably higher (*p <* 0.05), but there exhibited no remarkable difference between the Aerosol NAC group and the oral NAC group (*p >* 0.05) (Table 3). All results are shown in Figure 2.

**Table 3:** Comparison of clinical treatment efficacy among the three groups after 3 months of treatment.

Outcome	Control (*n* = 30)	Aerosol NAC (*n* = 30)	Oral NAC (*n* = 30)
Markedly successful response, *n* (%)	5 (16.7)	12 (40.0)	13 (43.3)
Successful response, *n* (%)	16 (53.3)	17 (56.7)	15 (50.0)
Treatment failure, *n* (%)	9 (30.0)	1 (3.3)	2 (6.7)
Treatment success rate, *n* (%)	21 (70.0)	29 (96.7)	28 (93.3)

Treatment success rate = (Markedly Successful Responses + Successful Responses)/Total Patients × 100%.

Bar chart showing the proportions of remarkably successful, successful, and unsuccessful treatment outcomes in patients receiving standard anti-tuberculosis therapy alone (Control group), standard therapy plus aerosolized N-acetylcysteine (Aerosol NAC group), and standard therapy plus oral N-acetylcysteine (Oral NAC group). Treatment efficacy was evaluated according to the Belknap (31). The treatment success rate was calculated as the proportion of remarkably successful and successful cases among the total number of patients in each group. Treatment success rates were 70.0% (21/30), 96.7% (29/30), and 93.3% (28/30) in the Control, Aerosol NAC, and Oral NAC groups, respectively. Both NAC-treated groups demonstrated significantly higher treatment success rates than the Control group (*p* < 0.05).

### 3.3 Negative conversion of sputum bacteria in three groups

**Figure 3:**
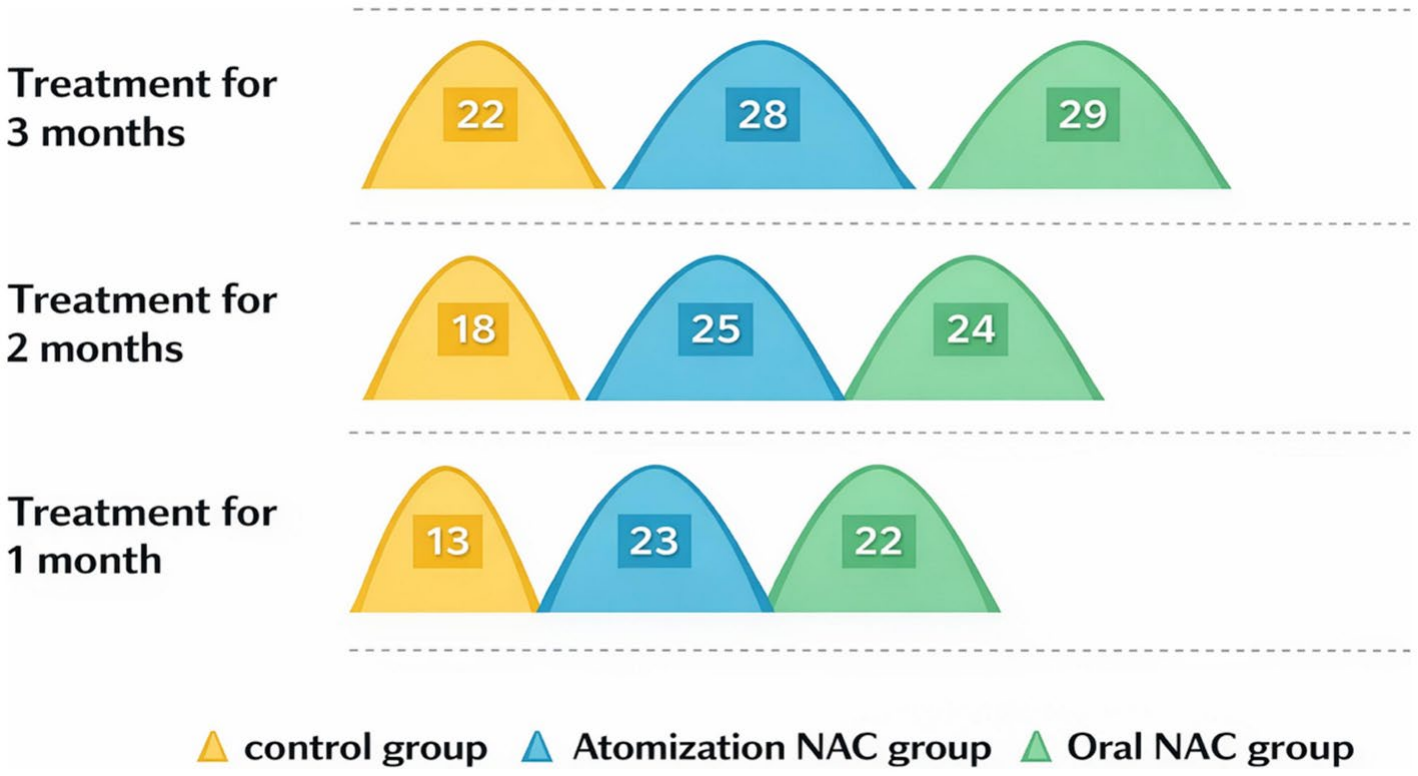
Sputum negative conversion in three groups.

After 1-, 2- and 3 months of treatment, the negative conversion rates of Aerosol NAC group were 76.67, 83.33 and 93.33%, respectively. The negative conversion rates of the NAC group were 73.33, 80 and 96.67%, respectively. The negative conversion rates of the control group were 43.33, 60 and 73.33%. The negative conversion rates of sputum bacteria in the Aerosol NAC group and the oral NAC group in each period after treatment were remarkably higher (*p <* 0.05), but there exhibited no remarkable difference (*p >* 0.05). All the results are shown in Figure 3.

### 3.4 Comparison of SGRQ scores among the three groups after treatment

Compared with the control group, the symptom score, activity score and daily life impact score of Aerosol NACgroup and oral NAC group were remarkably lower after 1-, 2- and 3-month treatment (*p <* 0.05). There exhibited no remarkable difference in the data between the Aerosol NAC group and the oral NAC group (*p <* 0.05). All results are shown in Table 4.

**Table 4:** SGRQ scores among the three groups after treatment (±s, points).

Group		*N*	Symptom score	Activity score	Daily life impact score
C Group	Treatment for 1 month	30	66.32 ± 5.72	75.43 ± 6.42	62.43 ± 4.68
	Treatment for 2 months		60.42 ± 6.21	69.23 ± 4.55	56.31 ± 5.53
	Treatment for 3 months		53.53 ± 7.42	62.03 ± 3.29	50.48 ± 6.71
Atomization NAC group	Treatment for 1 month	30	60.37 ± 6.54^*^	70.03 ± 6.51^*^	57.83 ± 5.63^*^
	Treatment for 2 months		52.51 ± 8.83^**^	62.39 ± 5.63^**^	48.17 ± 6.63^**^
	Treatment for 3 months		46.39 ± 8.35^***^	56.84 ± 8.83^***^	41.93 ± 5.84^***^
Oral NAC group	Treatment for 1 month	30	60.83 ± 6.31^*^	70.35 ± 6.69^*^	57.25 ± 6.72^*^
	Treatment for 2 months		51.89 ± 6.58^**^	63.04 ± 6.42^**^	47.93 ± 6.24^**^
	Treatment for 3 months		46.22 ± 8.74^***^	57.01 ± 7.37^***^	42.04 ± 7.08^***^

Compared with the control group: 1 month after treatment, *p < 0.05; 2 months after treatment, **p < 0.05; 3 months after treatment, ***p < 0.05.

### 3.5 Comparison of liver function indexes among the three groups

Compared with the control group, the levels of AST, ALT, TBIL and GGT in Aerosol NAC group and oral NAC group were remarkably lower after 1-, 2- and 3-month treatment (*p <* 0.05). There exhibited no remarkable difference between Aerosol NAC group and the oral NAC group (*p >* 0.05). All the results are shown in Table 5.

**Table 5:** The liver function indexes among the three groups (±s).

Groups		*N*	AST(U/L)	ALT(U/L)	TBIL(U/L)	GGT(U/L)
C Group	Treatment for 1 month	30	103.23 ± 40.52	121.34 ± 50.67	38.16 ± 12.09	120.87 ± 18.45
	Treatment for 2 months		83.42 ± 15.67	90.46 ± 44.25	24.51 ± 9.47	93.23 ± 14.07
	Treatment for 3 months		72.46 ± 14.53	85.54 ± 47.31	18.83 ± 8.73	84.93 ± 17.32
Aerosol NAC group	Treatment for 1 month	30	83.35 ± 38.84^*^	87.42 ± 51.08^*^	26.34 ± 10.83^*^	90.56 ± 10.09^*^
	Treatment for 2 months		65.42 ± 17.73^**^	62.33 ± 36.61^**^	19.93 ± 8.87^**^	74.32 ± 8.83^**^
	Treatment for 3 months		41.56 ± 13.38^***^	35.94 ± 32.58^***^	12.45 ± 5.56^***^	58.44 ± 10.51^***^
Oral NAC group	Treatment for 1 month	30	92.73 ± 37.52^*^	86.63 ± 47.73^*^	26.09 ± 9.87^*^	89.97 ± 9.88^*^
	Treatment for 2 months		74.06 ± 19.31^**^	62.08 ± 32.41^**^	18.94 ± 8.76^**^	73.74 ± 9.32^**^
	Treatment for 3 months		62.34 ± 15.53^***^	34.88 ± 30.98^***^	11.87 ± 5.73^***^	59.01 ± 8.79^***^

Compared with the control group: 1 month after treatment, *p < 0.05; 2 months after treatment, **p < 0.05; 3 months after treatment, ***p < 0.05.

### 3.6 The serum MDA, SOD, and GSH-XP levels among the three groups

The levels of serum GSH-PX and SOD in the aerosolized NAC group and the oral NAC group after 1-, 2- and 3 months of treatment were remarkably higher (*p <* 0.05), and the levels of MDA were remarkably lower (*p <* 0.05). There exhibited no remarkable difference in the data between the Aerosol NAC group and the oral NAC group (*p >* 0.05). All results are shown in Table 6.

**Table 6:** The serum MDA, SOD, and GSH-Px levels in three groups of patients (±s).

Group		*N*	MDA(μmol/L)	SOD(U/mL)	GSH-PX (μmol/L)
C Group	Treatment for 1 month	30	8.73 ± 1.56	164.69 ± 8.73	15.03 ± 0.61
	Treatment for 2 months		6.68 ± 1.25	203.41 ± 10.87	20.32 ± 1.32
	Treatment for 3 months		5.63 ± 1.32	245.51 ± 9.93	25.61 ± 1.51
Aerosol NAC group	Treatment for 1 month	30	6.26 ± 1.09	226.19 ± 10.08^*^	21.41 ± 1.09^*^
	Treatment for 2 months		5.04 ± 1.01	285.04 ± 11.81^**^	26.32 ± 0.93^**^
	Treatment for 3 months		3.46 ± 1.22	389.34 ± 20.07^***^	31.42 ± 1.28^***^
Oral NAC group	Treatment for 1 month	30	6.07 ± 1.04	225.32 ± 8.73^*^	20.93 ± 1.03^*^
	Treatment for 2 months		5.23 ± 0.97	283.18 ± 9.97^**^	26.45 ± 0.89^**^
	Treatment for 3 months		3.17 ± 0.89	404.45 ± 15.62^***^	30.93 ± 1.34^***^

Compared with the control group: 1 month after treatment, *p < 0.05; 2 months after treatment, **p < 0.05; 3 months after treatment, ***p < 0.05.

### 3.7 The serum inflammatory factors among three groups of patients

The levels of serum IFN-*γ*, PCT and IL-4 in the aerosolized NAC group and the oral NAC group after 1-, 2- and 3 months of treatment were remarkably lower (*p <* 0.05). There exhibited no remarkable difference in the data between the Aerosol NAC group and the oral NAC group (*p >* 0.05). All results are shown in Table 6.

### 3.8 Comparison of the incidence of adverse reactions among the three groups

In the control group, dizziness occurred in 1 case, diarrhoea in 3 cases, rash in 2 cases, fever in 4 cases, nausea and vomiting in 2 cases, and the incidence of adverse reactions was 40%. Diarrhoea, rash and nausea and vomiting occurred in 1 patient each in the Aerosol NAC group, and the incidence of adverse reactions was 10%. In the oral NAC group, 1 case had dizziness, 1 case had a fever, 1 case had nausea and vomiting, and the incidence of adverse reactions was 10%. Compared with the control group, the incidence of adverse reactions in the Aerosol NAC group and the oral NAC group was remarkably lower (*p* < 0.05), but there was no significant difference between the two NAC groups (*p* > 0.05) (Table 7). The detailed incidence of each type of adverse reaction is presented in Table 8.

**Table 7:** The serum inflammatory factors in three groups of patients(±s).

Group		*N*	IFN-γ(μg/L)	PCT (ng/mL)	IL-4(μg/L)
C Group	Treatment for 1 month	30	35.03 ± 7.56	5.03 ± 1.09	3.46 ± 0.71
	Treatment for 2 months		28.36 ± 6.87	3.02 ± 0.89	2.53 ± 0.45
	Treatment for 3 months		20.08 ± 7.73	1.46 ± 0.37	1.87 ± 0.32
Aerosol NAC group	Treatment for 1 month	30	26.74 ± 8.18^*^	3.84 ± 0.67^*^	2.93 ± 0.51^*^
	Treatment for 2 months		19.34 ± 7.73^**^	1.23 ± 0.41^**^	1.58 ± 0.47^**^
	Treatment for 3 months		13.42 ± 4.32^***^	0.46 ± 0.07^***^	0.61 ± 0.17^***^
Oral NAC group	Treatment for 1 month	30	26.32 ± 7.61^*^	3.66 ± 0.83^*^	2.79 ± 0.43^*^
	Treatment for 2 months		20.05 ± 8.03^**^	1.41 ± 0.52^**^	1.72 ± 0.51^**^
	Treatment for 3 months		18.87 ± 6.74^***^	0.53 ± 0.09^***^	0.58 ± 0.04^***^

Compared with the control group: 1 month after treatment, *p < 0.05; 2 months after treatment, **p < 0.05; 3 months after treatment, ***p < 0.05.

**Table 8:** Incidence of adverse reactions among the three groups [*n* (%)].

Group	Dizziness	Diarrhoea	Rash	Fever	Nausea/Vomiting	Overall incidence
Control (*n* = 30)	1 (3.3)	3 (10.0)	2 (6.7)	4 (13.3)	2 (6.7)	12 (40.0)
Aerosol NAC (*n* = 30)	0 (0)	1 (3.3)	1 (3.3)	0 (0)	1 (3.3)	3 (10.0)
Oral NAC (*n* = 30)	1 (3.3)	0 (0)	0 (0)	1 (3.3)	1 (3.3)	3 (10.0)

*p* < 0.05 for overall incidence in both NAC groups vs. control group; no statistically significant difference between aerosol NAC and oral NAC groups.

## 4 Discussion

Symptoms of pulmonary TB may include low fever, night sweats, fatigue, and fatigue-like symptoms. It is an infectious disease caused by MTB. At present, isoniazid, rifampicin, and pyrazinamide are commonly adopted when treating the disease, among which isoniazid is an antimicrobial agent, which has a strong bactericidal effect on MTB during the growth and reproduction period (17). Rifampicin is a first-line anti-TB drug with strong antibacterial activity and a strong killing effect on MTB in a growing period (18). Pyrazinamide can achieve high blood concentrations and wide distribution in a short period, penetrate phagocytes, affect MTB, elute amidases, and achieve an antibacterial effect (19). Ethambutol has a strong inhibitory effect on MTB and other mycobacteria, which can improve patients’ clinical symptoms such as cough and expectoration (20). However, long-term use is prone to resistance, which affects the antibacterial and bactericidal effect and has little effect on the inflammatory response, leading to limited long-term effects. NAC belongs to mucus dissolving agent, atomization inhalation can act on the trachea directly, which has the advantages of quick effect, high blood concentration and low toxicity and side effect. It can decompose mucin, and successfully reduce sputum viscosity, which is conducive to the smooth excretion of MTB. At the same time, it can also regulate the immune function of the body, promote the synthesis of complement and immune protein, alleviate inflammatory reactions, and shorten the course of disease (21, 22).

The results of this study indicated that in the control group, 5 people were remarkably successful, 16 people were successful, 9 people were unsuccessful, and the success rate was 70%. In the Aerosol NAC group, 12 people were remarkably successful, 17 people were successful, 1 person was unsuccessful, and the success rate was 96.67% (22). In the oral NAC group, 13 people were remarkably successful, 15 people were successful, and 2 people were unsuccessful, and the success rate was 93.33%. Compared with the control group, the success rates of the Aerosol NAC group and the oral NAC group were remarkably higher, but there exhibited no remarkable difference between the Aerosol NAC group and the oral NAC group (23). Compared with the control group, the symptom scores, activity scores and daily life impact scores of the Aerosol NAC group and the oral NAC group after 1-, 2- and 3 months of treatment were remarkably lower. There exhibited no remarkable difference in the data between the Aerosol NAC group and the oral NAC group. This suggests that treatment with conventional anti-TB drugs in combination with acetylcysteine provides faster clinical relief and is equally successful when administered orally or by nebulization (24). It is suggested that NAC can improve the physical and chemical properties of the sputum of pulmonary TB. The reason is that the sulfhydryl group in the molecular structure of acetylcysteine breaks the disulfide bond between mucin molecular complexes, reduces the viscosity of sputum and makes sputum easy to cough up. After 1-, 2- and 3 months of treatment, the negative conversion rates of Aerosol NAC group were 76.67, 83.33 and 93.33%, respectively. The negative conversion rates of the NAC group were 73.33, 80 and 96.67%, respectively. The negative conversion rates of the control group were 43.33, 60 and 73.33%, respectively. The negative conversion rates of sputum bacteria in the Aerosol NAC group and the oral NAC group in each period after treatment were remarkably higher, but there exhibited no remarkable difference. The results indicated that acetylcysteine could remarkably shorten the time to negative sputum, increase the rate of early negative sputum, promote the absorption of lesions, and reduce the transmission of TB. The efficacy of acetylcysteine when treating newly diagnosed TB not only improves clinical symptoms but also fundamentally improves the patient’s condition and prevents disease recurrence (24, 25).

At present, the commonly used anti-TB drugs need to be metabolized through the liver, which can lead to liver toxicity and drug-induced liver injury (DILI). Domestic scholars pointed out that the incidence of DILI in China indicated a remarkable increasing trend (23). DILI accounts for 15% of drug-responsive diseases, of which more than 20% of acute liver injury is caused by DILI. In a foreign study, some scholars have confirmed that hepatotoxicity exists in 1100 known drugs on the market in the world (24). In foreign countries, many scholars have used NAC to treat patients with DILI (25). It has been confirmed that NAC can promote the recovery of liver function and improve the prognosis. The results of this study indicated that compared with the control group, the levels of AST, ALT, TBIL and GGT were remarkably lower in the aerosolized NAC group and the oral NAC group after 1-, 2- and 3 months of treatment. There exhibited no remarkable difference in the data between the Aerosol NAC group and the oral NAC group (20, 21). As a result of different methods of administering acetylcysteine, it is suggested that liver function can be successfully recovered in patients with pulmonary tuberculosis by combining it with conventional anti-TB drugs. NAC is formed by L-cysteine plus acetyl, which can promote the synthesis of glutathione and protect the liver (21). At the same time, NAC also contains active vegetable groups, which can resist tissue oxidative damage caused by different causes and play a role in protecting the liver. In this study, the combination achieved better results and we analyzed that the combination of the two drugs could protect the liver of patients with DILI caused by anti-TB drugs (20, 22–24).

Oxidative stress can attach importance to the occurrence and development of liver disease. At the same time, inflammation also affects the aggregation of neutrophils and the repair of hepatocytes. Isoniazid, rifampicin, and pyrazinamide are commonly used drugs when treating pulmonary TB^27^. Long-term studies have found that isoniazid treatment of DILI may be related to metabolic enzymes CYP2E1, N-acetyltransferase and glutathione S-transferase. The mechanism of DILI induced by rifampicin may be related to peroxidase activating the gamma signal pathway and glutathione transferase arachidonic acid. The mechanism of DILI induced by pyrazinamide may be related to oxidative stress, hepatocyte apoptosis, bile acid synthesis or dysfunction (26). It is reported that inhaled NAC is successful when treating elderly patients with mechanical ventilation of ARDS. It can reduce lung injury and improve blood gas and blood oxygen by regulating the indexes of IL-4, IL-10, TNF- *α* and MDA in bronchoalveolar lavage fluid (BALF). SOD and MDA are commonly used in the clinical observation of oxidative stress. IFN- *γ*, PCT and IL-4 are all important indicators to judge the degree of infection and inflammatory reaction. IFN-γ is an immunomodulatory factor in TB patients. Proliferation of MTB stimulates the immune system to release IFN-γ, resulting in abnormally high levels of IFN-γ, which can reflect the extent of TB infection. PCT is a procalcitonin peptide, and its expression level is low in a healthy state (24). When the body is invaded by pathogenic bacteria, the level of serum PCT increases rapidly, which is an important diagnostic index of respiratory tract infection. IL-4 can attach importance to the inflammatory response. When the body has an inflammatory response, its level tends to rise, which can detect airway inflammation in the body (26, 27). The results of this study indicated that the levels of serum GSH-Px and SOD in the aerosolized NAC group and the oral NAC group after 1-, 2- and 3 months of treatment were remarkably higher, and the levels of MDA were remarkably lower compared with the control group. There exhibited no remarkable difference in the data between the Aerosol NAC group and the oral NAC group. The levels of serum IFN-*γ*, PCT and IL-4 in the aerosolized NAC group and the oral NAC group after 1-, 2-, and 3 months of treatment were remarkably lower. There exhibited no remarkable difference in the data between the Aerosol NAC group and the oral NAC group. It can be seen that combination therapy can better resist oxidative stress and inflammatory response in patients with DILI caused by TB drugs (25). NAC can reduce the activity of mitochondrial adenosine triphosphate activating enzyme in liver cells, improve the energy supply of liver cells, reduce the consumption of SOD in the liver, and reduce the degree of oxidation reaction, thereby reducing the level of MDA. NAC also has the effect of anti-inflammatory and promotes the production of glutathione, which can stimulate the activity of growth-related pathways and promote the regeneration and repair of liver cells (26). Finally, in the observation of adverse reactions after treatment of patients, it was found that 1 person in the control group had dizziness, 3 people of diarrhoea, 2 people of rash, 4 people of fever, 2 people of nausea and vomiting, and the incidence of adverse reactions was 40%. Diarrhoea, rash and nausea and vomiting occurred in 1 patient each in the Aerosol NAC group, and the incidence of adverse reactions was 10%. In the oral NAC group, 1 person had dizziness, 1 person had a fever, 1 person had nausea and vomiting, and the incidence of adverse reactions was 10% (28–30).

The open-label design may have introduced performance or detection bias, particularly for subjective outcomes such as symptom scores. However, the use of objective endpoints (sputum conversion, laboratory biomarkers, and CT lesion absorption assessed by blinded personnel) and the nearly identical results between the two NAC arms mitigate this concern. The sample size was not formally calculated *a priori*. Although post-hoc power was adequate for the primary endpoint, the study may have been underpowered for some secondary outcomes or subgroup analyses.

Several limitations of this study should be acknowledged. First, this was an open-label trial; double-blinding was not feasible due to the different routes of administration. Although objective laboratory and imaging endpoints were assessed by blinded personnel, subjective symptom scores may carry some detection bias. Second, the trial was not prospectively registered in a public clinical trial registry, which is a requirement of the ICMJE. Third, baseline (pre-treatment) liver function values were not systematically recorded for all patients; therefore, we cannot completely exclude the possibility that groups differed at baseline. Fourth, the sample size was not formally calculated *a priori* and the study was conducted at a single center, limiting generalizability. Fifth, follow-up was limited to 3 months; long-term efficacy, relapse rates, and durability of the antioxidant/anti-inflammatory effects of NAC were not assessed. Sixth, although both NAC regimens showed clear benefits, the study was not powered to detect small differences between aerosol and oral NAC. Despite these limitations, the study provides valuable mechanistic and clinical data supporting the use of NAC as adjunctive therapy in newly diagnosed pulmonary tuberculosis. Future multicenter, double-blind, placebo-controlled trials with larger samples, baseline biomarker collection, prospective registration, and longer follow-up are warranted.

## 5 Conclusion

Conventional anti-tuberculosis drugs combined with either aerosol inhalation or oral N-acetylcysteine significantly improved clinical efficacy, accelerated sputum negative conversion, alleviated symptoms, protected liver function, reduced oxidative stress, and attenuated systemic inflammation in patients with newly diagnosed pulmonary tuberculosis, with a favorable safety profile and low incidence of adverse reactions. Both routes of NAC administration were similarly effective. These findings support the addition of N-acetylcysteine as a safe and beneficial adjunct to standard HRZE therapy in newly diagnosed drug-susceptible pulmonary TB. Larger, multicenter, randomized controlled trials with longer follow-up are needed to confirm these results and to determine the optimal dose, duration, and route of NAC.

## Data Availability

The original contributions presented in the study are included in the article/supplementary material, further inquiries can be directed to the corresponding author.
